# CD36 and Its Role in Obesity

**DOI:** 10.1111/obr.70039

**Published:** 2025-12-04

**Authors:** Nahuel Garcia, Maiken Mellergaard, Carlos Salomon, Pilar Sepulveda, Peter Kristensen, Aase Handberg

**Affiliations:** ^1^ GECORP Buenos Aires Argentina; ^2^ Department of Clinical Biochemistry Aalborg University Hospital Aalborg Denmark; ^3^ Department of Clinical Medicine, Faculty of Medicine Aalborg University Aalborg Denmark; ^4^ Translational Extracellular Vesicles in Obstetrics and Gynae‐Oncology Group University of Queensland Brisbane Queensland Australia; ^5^ Regenerative Medicine and Heart Transplantation Unit Instituto de Investigación Sanitaria La Fe Valencia Spain; ^6^ Department of Pathology University of Valencia Valencia Spain; ^7^ Department of Chemistry and Bioscience, Faculty of Engineering and Science Aalborg University Aalborg Denmark

**Keywords:** CD36, ectopic fat, obesity

## Abstract

Obesity is understood as a condition driven by interactions between genetics and environmental factors. The role of CD36 in the regulation of lipid metabolism and ectopic fat accumulation emerges as a key area of interest. This review presents CD36 not only as a crucial facilitator of fatty acid uptake but also as a regulator of how and where excess lipids are stored. Ectopic fat accumulation—lipid deposition in non‐adipose tissues such as the liver, muscle, and pancreas—is linked to obesity‐related complications, including metabolic dysfunction‐associated steatotic liver disease (MASLD) and cardiovascular risk. Through CD36, tissues that normally play minor roles in lipid storage become overloaded, leading to metabolic dysfunction. We offer a fresh perspective on the adipose tissue expandability hypothesis, positioning CD36 as a regulator of adipose tissue's capacity to store lipids. Possibly, once adipose tissue reaches its expansion limit, CD36‐mediated mechanisms drive the spillover of lipids into ectopic sites, exacerbating obesity complications. This insight offers a transformative view of CD36 as a player in the metabolic tipping point between healthy fat storage and pathogenic fat deposition. The connection between CD36 and extracellular vesicles (EVs) hints at a broader network of inter‐tissue communication that could further amplify ectopic fat accumulation. Finally, we list evidence showing how CD36 genetics are related to the predisposition to develop and manage obesity. By understanding the role of CD36 in fat storage regulation, new personalized therapeutic strategies may emerge, targeting its pathways to prevent or reverse the metabolic damage caused by ectopic fat.

## 1 Introduction

The escalating global obesity prevalence constitutes a widespread concern that arises from the correlation between elevated body mass index (BMI) and increased occurrence of cardiovascular disease, diabetes, and hypertension [1]. The multifaceted mechanisms of the obesity pandemic are intricate and manifold. Proposed causes range from genetic predisposition [1] to insufficient sleep [2], climate change impacts [3], excess energy intake (especially associated with the consumption of fast food) [4], and factors such as unfavorable neighborhood environments [5], antidepressant usage [6], and social influence through associations with overweight peers [7]. It is plausible that the comprehensive increase in obesity reflects the cumulative impact of numerous factors, each exerting a relatively modest individual influence. Genetics, together with dietary habits and nutritional changes, is highly likely to play a central role in this complex interplay. The consumption of an unbalanced and unhealthy diet and subsequent lipid accumulation are implicated in the development of overweight and obesity [8]. Maintaining a balanced fat intake is crucial for human health, as it not only supplies essential fatty acids (FAs) and fat‐soluble vitamins but also plays a key role in regulating satiety and energy homeostasis. Ingested fats undergo hydrolysis within the fat digestive system to yield FAs. Various FA transporting systems, including CD36, fatty acid‐binding proteins (FABPs), and fatty acid transport proteins (FATPs), facilitate the translocation of long‐chain FAs (LCFAs) into intestinal epithelial cells and extraintestinal tissues such as the liver, adipose tissue (AT), and muscle [9]. Notably, emerging evidence suggests that FA transporters, like CD36, serve as sensors and regulators of FA homeostasis [10].

The study of FA metabolism has evolved over the 20th century and continues to be a dynamic field of research in the 21st century with ongoing discoveries contributing to our understanding of the role of FAs in cellular function, energy metabolism, and various physiological processes. Cells acquire FAs through two main sources: the lipoprotein triglyceride hydrolysis catalyzed by lipoprotein lipase (LPL) and unesterified FAs transport associated with albumin [9]. The association between CD36 as an FA transporter and obesity lies in the ability of CD36 to modulate lipid uptake, metabolism, and storage [11]. In this review, we will focus on the uptake of free FAs (FFAs) by CD36 and its role in the development of obesity and related complications, with a special focus on ectopic fat accumulation.

## 2 CD36 Genetics, Structure, and Function

CD36, also known as cluster of differentiation 36, is an 80 kD integral membrane protein heavily glycosylated and serves as a scavenger receptor crucial for the high‐affinity uptake of LCFAs in various tissues. Its role in facilitating cellular FA uptake was initially identified in 1993 [12] and has been strongly supported by evidence from studies involving CD36‐deficient rodents and humans [9]. The CD36 gene, situated on chromosome 7q21.11 and spanning approximately 77 kb, exhibits extensive alternative splicing with at least 20 transcript variants identified. These include 16 coding mRNA, 1 non‐coding mRNA, and 3 predicted mRNA, derived through automated computational analysis of CD36 mRNA and expressed sequence tag (EST) data. The gene consists of 19 exons with up to 15 exons spliced in a single mRNA variant of which 12 are coding. Notably, major transcript variants (isoform 1 and 3) feature non‐coding regions in exon 1, 2, and the 5′‐end of exon 3 [13].

**FIGURE 1:**
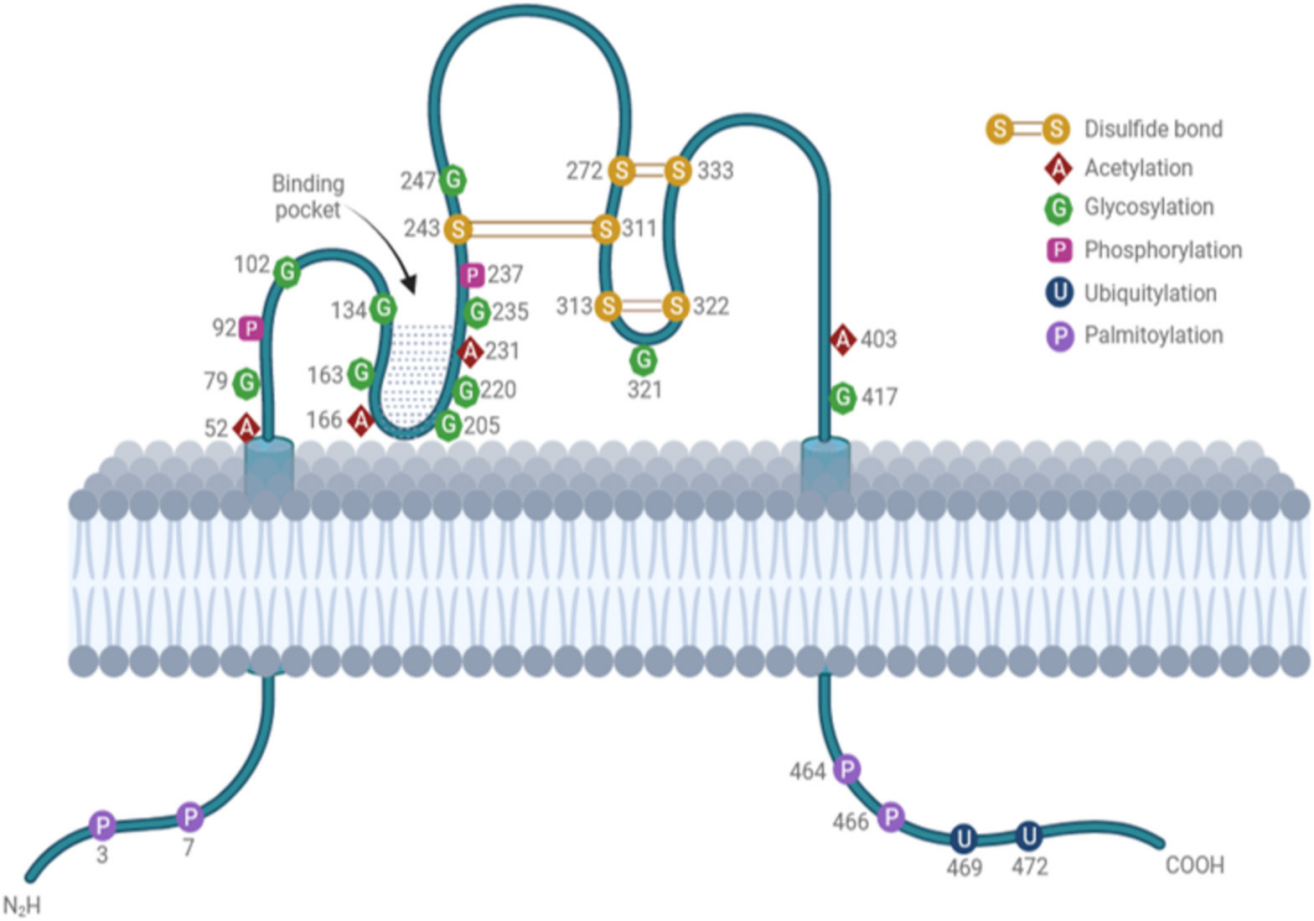
CD36 structure and post‐transcriptional modifications scheme.

CD36 is widely expressed in various cell types, including platelets, immune cells, hepatocytes, adipocytes, myocytes, enterocytes, enteroendocrine cells, retinal and mammary epithelial cells, and microvascular endothelial cells. The CD36 transmembrane protein, crucial for signal transduction, comprises an N‐terminal and a C‐terminal cytoplasmic tail, Src‐family tyrosine kinases, two transmembranes (Aa8–29 and Aa440–461), and a large extracellular loop (Aa30–439) with hairpin‐like membrane topology (Figure 1). The heavily glycosylated extracellular domain, pivotal for CD36 membrane recruitment, possesses three disulfide bridges in the carboxyl‐terminal half. A hydrophobic amino acid stretch (186–204) may form a binding pocket for lipid ligands, contributing to its function [14]. The cytoplasmic tails of the protein are short but active in signal transduction through interactions with tyrosine kinases. CD36 undergoes various posttranslational modifications, such as phosphorylation, glycosylation, palmitoylation, acetylation, and ubiquitination, which influence its expression levels, trafficking, and overall functions [14].

The CD36 gene exhibits diverse nucleotide variants, totaling 30,703 in the single nucleotide polymorphism database (dbSNP), comprising 26,531 single nucleotide variants (SNVs) and 4172 multiple nucleotide variants (MNVs) or deletion/insertion variants (DELINS). Primarily located in non‐coding regions, most variants do not induce amino acid or functional changes. However, a subset, including 590 missense mutations in the coding region or flanking splice sites, can impact CD36 protein function. Additionally, certain non‐coding region variants are associated with protein expression levels and clinical symptoms [13].

The structure–function understanding of CD36 is still incomplete, but some advancements have shed light on its characteristics and two main mechanisms are currently accepted for different cell types such as muscle cells and adipocytes (described below as model 1 and model 2, respectively).

### 2.1 CD36 FAs Uptake—Model 1

**FIGURE 2:**
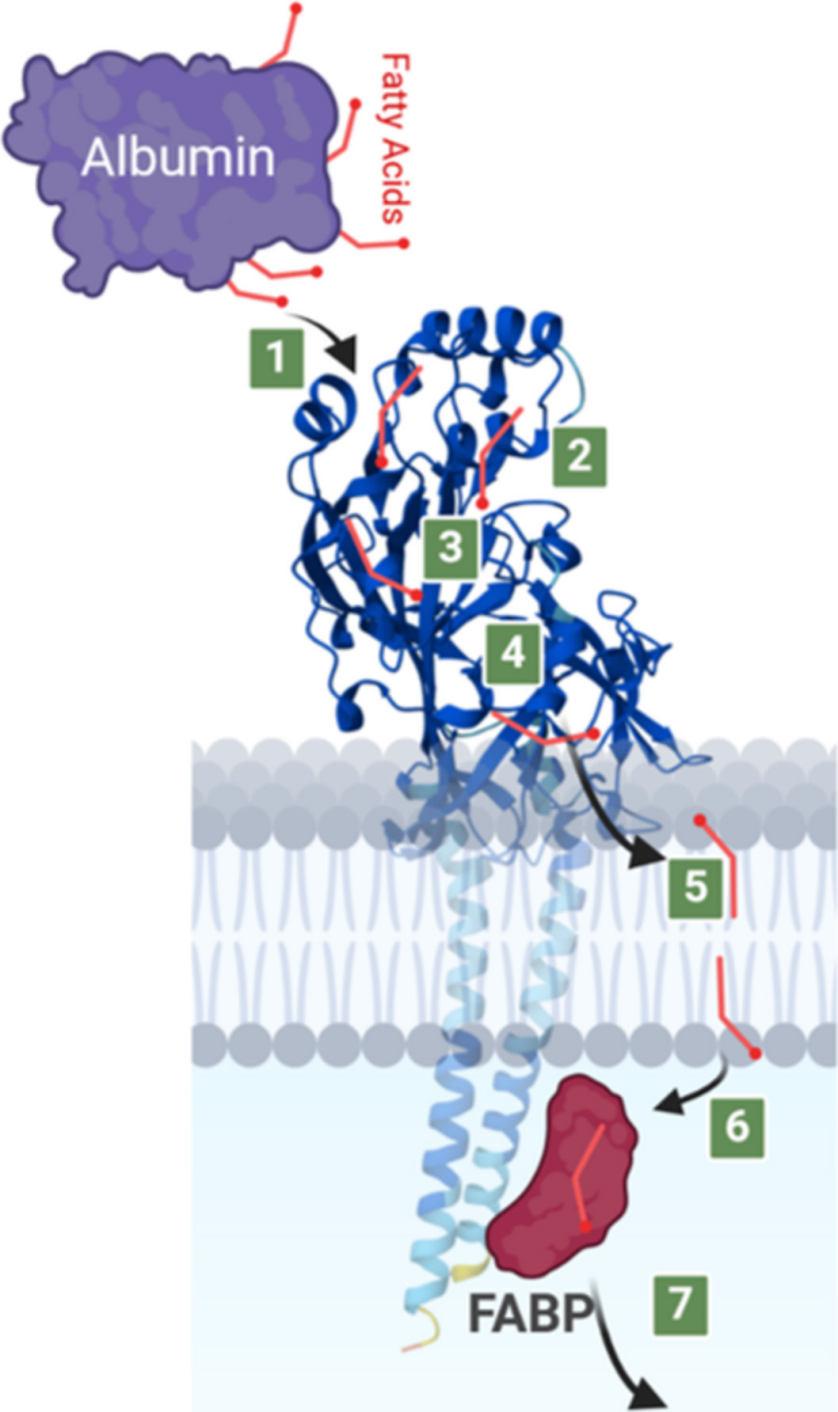
Model 1. Illustration depicting the progressive steps required for cellular uptake of LCFAs includes the following: 1. The liberation of FAs from interstitial albumin. 2. Encapsulation within the hydrophobic core of CD36. 3. The navigation of the FA through the interior of CD36 ectodomain, facilitating its movement across the unstirred water layer to reach the plasma membrane. 4. The release of the FA from CD36 into the external layer of the membrane. 5. The ‘flip‐flop’ translocation of individual FAs across the membrane. 6. The detachment of FAs from the bilayer's inner leaflet and their attachment to the core of cytoplasmic fatty acid‐binding protein (FABPc), which itself is tethered to the cytoplasmic region of CD36. 7. The movement of the fatty acid–FABPc complex through the cytosol to areas involved in the intracellular breakdown and utilization of FAs.

Consensus has recently been reached regarding the molecular mechanism of LCFA uptake by muscle cells and highlights the role of membrane‐associated proteins like CD36 that facilitate FA movement within the plasma membrane through passive diffusion (‘flip–flop’)(Figure 2). As such, CD36 facilitates desorption at the inner membrane and subsequently binds to cytoplasmic fatty acid‐binding protein (FABP). The presence of CD36 accelerates the overall rate of FA uptake or release from adipocytes [15]. In the heart, CD36 emerges as the primary facilitator of FA uptake and is crucial for transendothelial FA transport and uptake into cardiomyocytes [16, 17].

Short‐term regulation of cellular FA uptake involves the continuous recycling of CD36 between endosomes and the sarcolemma. Insulin or muscle contraction triggers the translocation of CD36 to the sarcolemma, increasing the rate of FA uptake, with immediate internalization upon trigger removal [18]. This regulatory mechanism bears similarities to the regulation of myocardial glucose uptake through the recycling of glucose transporter‐4 (GLUT4) [19].

### 2.2 CD36 FAs Uptake—Model 2

**FIGURE 3:**
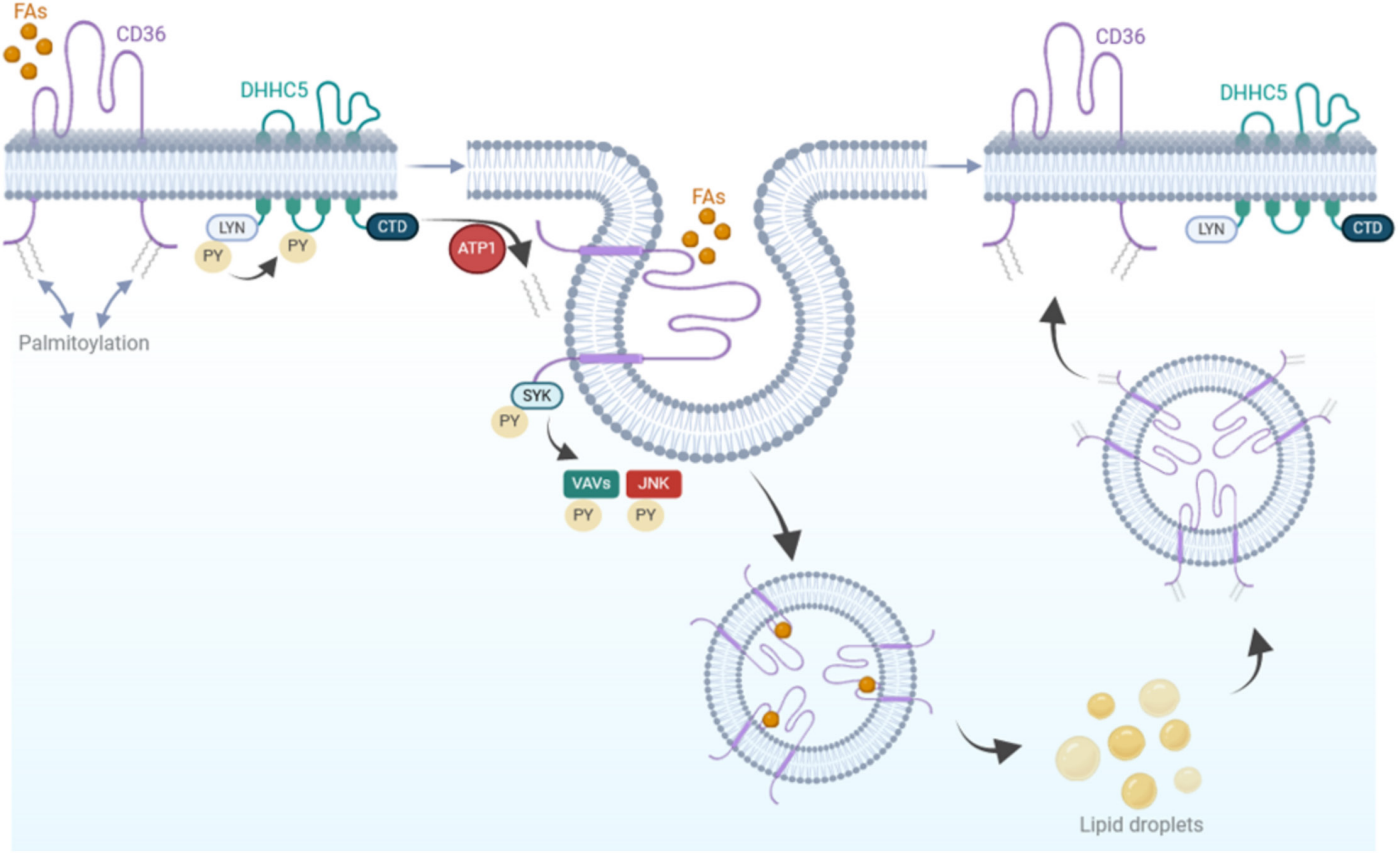
Model 2. Palmitoylation facilitates the positioning of CD36 on the cellular membrane for the purpose of engaging with FAs. This interaction with FAs subsequently triggers the activation of the downstream kinase LYN associated with CD36, leading to the phosphorylation of palmitoyl acyltransferase. This phosphorylation event then instigates the subsequent depalmitoylation process targeting CD36. Once depalmitoylated, CD36 recruits the tyrosine kinase SYK to initiate the phosphorylation of the JNK and VAV kinases, thereby triggering caveolar endocytosis and facilitating the transfer of FAs to lipid droplets. Upon successful delivery, CD36 undergoes repalmitoylation and is subsequently recycled back to the membrane, ready for another cycle of FA transport. C‐terminal domain (CTD). Phosphorylation (PY). Palmitoyl acyltransferase (DHHC5). Depalmitoylase (ATP1).

The regulation of CD36 expression and its cellular localization in adipocytes involves a tightly controlled process of palmitoylation domains, orchestrated by palmitoyl acyltransferase [20, 21]. The modification by palmitoylation facilitates the translocation of CD36 to the cell membrane, enabling its interaction with FAs [22]. For CD36 to facilitate the transport of FAs into the cell, it must undergo depalmitoylation (Figure 3). This depalmitoylation process, crucial for endocytosis, is initiated by FA binding, which activates CD36 downstream of Lck/yes‐related protein tyrosine kinase (LYN). Subsequently, LYN phosphorylates the palmitoyl acyltransferase DHHC5, leading to depalmitoylation of CD36 by the depalmitoylase ATP1. Ultimately, the depalmitoylated CD36 recruits the tyrosine kinase SYK/JNK/VAV pathway, instigating caveolar endocytosis [22]. The predominant cellular localization of CD36 is within a concave structure on the cell membrane, forming a hole‐like configuration. Its FA‐binding pocket is positioned on the cell surface, allowing for the capture and transfer of FAs to lipid droplets. Consequently, CD36‐mediated caveolae endocytosis effectively facilitates FA uptake. In cases of endocytic dysfunction, CD36‐dependent lipid droplet growth in adipocytes is impeded, preventing high‐fat diet (HFD)‐induced weight gain in mice. It is noteworthy that blocking CD36 in the depalmitoylated state does not eliminate CD36‐dependent FA uptake, suggesting that the dynamic CD36 palmitoylation‐depalmitoylation cycle may play a role in caveolae‐dependent CD36 internalization and the reorganization of the cytoskeleton for the delivery of FAs [22].

Overall, Model 1 (flip‐flop mechanisms) has been shown in adipocytes and cardiomyocytes, and Model 2 (caveolar endocytosis) has only been studied in adipocytes.

## 3 CD36 in Metabolic Homeostasis

One of the first insights into the role of CD36 in whole‐body metabolic homeostasis occurred in 1999, when Aitman et al., showed that the absence of CD36 contributes to insulin resistance (IR), impaired FA metabolism, and elevated levels of triglycerides in the spontaneously hypertensive rat [23]. CD36 presence throughout the digestive tract, including taste bud cells in the tongue, stomach, small intestine, and colon, emphasizes its role in chylomicron production. Within the digestive tract, CD36 facilitates the uptake of FAs and cholesterol by proximal enterocytes, optimizing chylomicron production [24, 25]. Despite defects in lipid uptake and chylomicron formation in CD36 deficiency, overall intestinal lipid absorption is minimally affected, except for very LCFAs. CD36 may also contribute to the intracellular trafficking of fats and cholesterol for packaging into lipoproteins, highlighting its significance in ensuring efficient gut lipid absorption and subsequent lipid metabolism. CD36 impact extends to fat taste perception through binding and uptake of FAs in taste bud cells, contributing to fat preference regulation [25].

Glatz and colleagues highlighted that the transport and oxidation processes of LCFAs within skeletal muscle are upregulated due to the enhanced expression, protein synthesis, and relocation of CD36 [26]. Furthermore, it was observed that FAs induce an increase in the expression and synthesis of CD36 through the activation of transcription factors known as peroxisome proliferator‐activated receptors (PPARs), which are notably responsive to FAs [27]. This feedback mechanism suggests that CD36 primarily fulfills the energy requirements derived from lipid sources, particularly in metabolically active tissues like skeletal muscle. A significant correlation has been established between the activation of AMP‐activated protein kinase (AMPK), the translocation of CD36 to the plasma membrane, and the subsequent augmentation in the uptake and oxidation of FAs [28]. Notably, the transcription, translation, protein abundance, and relocation of CD36 are delicately regulated by dietary intake [29].

CD36 activity has also been observed to govern the operation of AMPK in orchestrating skeletal muscle absorption of FAs alongside their subsequent utilization within mitochondria [28]. Conversely, there have been reports of AMPK boosting the absorption of intestinal FAs by escalating the expression of CD36 protein and prompting its relocation to the membrane concurrently [30]. These discoveries suggest a fluid interaction between CD36 and AMPK.

### 3.1 CD36 in Inflammation and IR in Obesity

In obesity, systemic low‐grade inflammation (e.g., circulating TNF‐α, IL‐6, adipokines, endotoxemia) and tissue‐intrinsic inflammatory signals (macrophage infiltration, hypoxia/ER stress, lipid overload, and DAMPs) converge to regulate CD36 across organs [31, 32, 33]. At the systemic level, cytokine and danger‐signal exposure enhance CD36 expression and/or surface availability via canonical inflammatory pathways, thereby facilitating uptake of fatty acids and modified lipids and amplifying lipotoxic stress [32, 34]. Locally, adipose‐tissue inflammation increases CD36 in adipocytes and resident immune cells; the liver exhibits CD36‐linked steatosis and Kupffer‐cell activation; skeletal muscle shows altered CD36 trafficking and lipid handling; the endothelium upregulates CD36 in pro‐atherogenic contexts; and pancreatic islets display β‐cell CD36 changes that intersect with glucolipotoxicity [31, 32, 33, 35]. Together, these systemic and local inflammatory cues shape CD36‐mediated lipid flux and ectopic fat deposition.

Chronic low‐grade inflammation is a characteristic feature of obesity, contributing to IR and metabolic dysfunction [31]. Excessive weight gain triggers the elevation of CD36 levels in early‐stage adipocytes, fostering inflammatory responses by disturbing the balance of calcium within lysosomes and impairing their functionality. This aberrant increase in CD36 expression within early‐stage adipocytes is implicated in the initiation of inflammation within ATs [36]. CD36 as a FA receptor is central for the regulation of immune cell activity by directing immunometabolic activity and thus, inflammation via immunity (recently reviewed by Chen et al.) [32]. While these topics exceed the purpose of this review, several works have studied the relationship between CD36, inflammation, and IR [32, 33, 34, 35, 36].

Insulin acutely mobilizes CD36 from endosomal stores to the sarcolemma, increasing LCFA uptake; removal of the stimulus reverses this within minutes, a process analogous to GLUT4 recycling [18, 19]. Muscle contraction/AMPK activation elicits a similar translocation that couples uptake to β‐oxidation [28, 37]. In insulin‐resistant states, myotubes show increased CD36 cycling and cell‐surface retention, favoring lipid accumulation [30, 38]. Across tissues, hyperinsulinemia can upregulate CD36 expression and lipid influx; in hepatocytes, CD36 also promotes SREBP1 processing via INSIG2 to drive de novo lipogenesis, while hepatocyte‐specific CD36 deletion attenuates steatosis and improves insulin sensitivity [39, 40, 41]. In adipose tissue—but not skeletal muscle—insulin enhances LPL activity, augmenting FA delivery to CD36‐positive cells and amplifying this axis [42]. CD36 has been noted to engage with the insulin receptor to facilitate tyrosine phosphorylation of this receptor and amplify downstream insulin signaling—a process that is hindered by FAs [38]. By characterizing CD36 transcript expression in adipose and muscle tissues of individuals with obesity, Pietka et al. emphasize CD36 involvement in FA regulation and insulin sensitivity. Various CD36 transcript variants exhibit distinct effects on tissue CD36 levels, thereby impacting FA balance and insulin responsiveness [43].

### 3.2 CD36 in Sensory Perception

CD36 is expressed on ~12%–15% of taste bud cells in the circumvallate papillae and mediates long‐chain FA sensing; LCFAs evoke CD36‐dependent Ca^2+^ signals, neurotransmitter release, and activation of the gustatory pathway, triggering cephalic‐phase digestive secretions [44, 45, 46, 47]. Functionally, lingual CD36 increases spontaneous fat preference and intake [46, 47]. In contrast, intestinal lipid sensing engages a satiety brake: duodenal fat stimulates the oleoylethanolamide (OEA) pathway, and CD36 deficiency abolishes OEA production, linking enteric CD36 to meal termination [48]. Human genetic variation at CD36 associates with differences in fat‐taste sensitivity, with downstream links to adiposity traits in some cohorts [49, 50, 51, 52, 53]. Together, these data support a bidirectional model in which CD36‐mediated fat sensing shapes appetite, enhancing orosensory fat preference while promoting intestinal feedback that limits meal size, thereby influencing energy intake and obesity risk. Mechanistically, gut AMPK can increase enterocyte FA uptake by upregulating and translocating CD36, suggesting a tunable intestinal CD36‐satiety axis [30]. Beyond sensory tissues, CD36‐facilitated FA uptake in adipose inhibits leptin production and signaling, potentially weakening anorexigenic feedback and further connecting CD36 activity to appetite control [11].

In summary, CD36 operates at the interface of sensory detection and metabolic signaling to regulate fat preference, satiety, and ultimately caloric intake, providing a mechanistic bridge from sensory perception to obesity.

### 3.3 CD36 as a Regulator of Circadian Rhythms and Senescence

The aging process is closely related to the regulation of the circadian rhythm [54]. Moreover, aging and circadian rhythms are related to obesity [2, 55]. Interestingly, recent studies have linked aging and circadian protein homeostasis with CD36. The rhythmic expression of CD36 in the mouse liver autonomously influences the diurnal oscillations of the hepatic clock and the maintenance of glucose homeostasis. CD36 plays a pivotal role in regulating the circadian oscillator, and its insufficiency could lead to disruptions in the liver clock. This disruption may exacerbate the disturbance in glucose homeostasis, thereby contributing to the amplification and progression of metabolic disorders [56].

The process of cellular senescence, which marks the irreversible cessation of cell division, is closely linked to aging and obesity. A senescence‐like phenotype is induced with the increased expression of CD36 [57]. In mice, CD36 mediates the senescence‐associated secretory phenotype that arises in aged muscle cells. Moreover, the conditioned medium derived from human fibroblasts overexpressing CD36 alone demonstrates the capacity to prompt a senescence‐like phenotype in actively dividing young cells [58]. Acyltransferase DHHC5 is a critical player for FAs uptake by regulating the palmitoylation state of CD36. Interestingly, recent work has identified DHHC5‐mediated palmitoylation activity as a fundamental process through which aging accelerates the decline in autophagy [59].

## 4 CD36 in Ectopic Fat Accumulation

**FIGURE 4:**
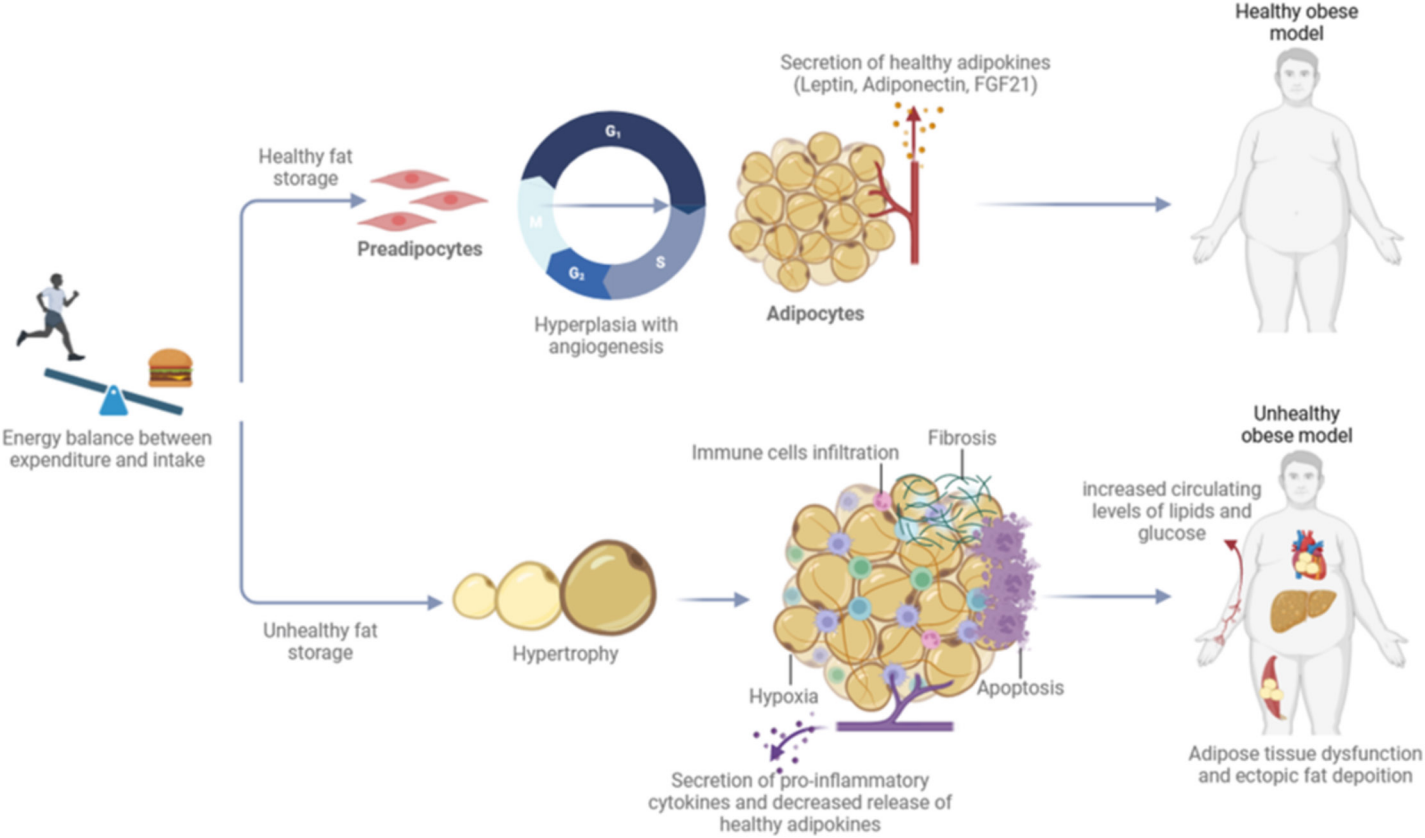
Healthy vs. unhealthy fat expansion models. When calorie intake exceeds energy expenditure the metabolic fuel oversupply is stored in/as fat tissue. If this energy imbalance is prolonged in time, the AT expands in two possible ways: via differentiation of local tissue progenitors to generate fresh adipocytes (hyperplasia) or via the expansion of pre‐existing adipocytes (hypertrophic growth). Expansion of AT through increased cell numbers, known as hyperplasia, is regarded as a beneficial and adaptive process by sustaining adequate vascular supply and regulate levels of adiponectin, an insulin‐sensitizing and anti‐inflammatory hormone, along with other adipokines involved in metabolic modulation. Conversely, hypertrophy of adipocytes, characterized by their enlarged size, is linked to increased cellular hypoxia. AT exhibits an inadequate hypoxic response including promptly upregulation of pro‐fibrotic genes, promoting tissue fibrosis. In some instances, hypoxic adipocytes undergo necrosis, triggering infiltration by immune cells and instigating tissue inflammation. These cumulative effects impair AT functionality, culminating in sustained elevation of circulating nutrients (glucose and lipids) and accelerating the onset of metabolic disorders. Furthermore, this dysregulated AT function contributes to the ectopic deposition of lipids in tissues such as muscle and liver.

Adipocytes, specialized for the accumulation of surplus metabolic fuels in the form of energy stores, fulfill diverse physiological roles, encompassing insulation, thermogenesis, mechanical support, and organ safeguarding. AT, once considered a passive reservoir of energy, is now acknowledged as a dynamic endocrine organ that releases adipokines and influences systemic metabolism [60]. In cases of metabolic dysregulation (characterized by reduced insulin sensitivity, impaired metabolic flexibility, and adipose tissue dysfunction) under chronic positive energy balance associated with obesity, adipocytes exhibit impaired responsiveness to signals, resulting in elevated levels of glucose and lipids in circulation. Dysfunctional adipocytes manifest altered transcriptional programs, elevated collagen synthesis, and increased adipocyte necrosis, contributing to the release of pro‐inflammatory cytokines [61]. The expansion of AT is primarily governed by two mechanisms: hypertrophy (enlargement of existing adipocytes) and hyperplasia (formation of new adipocytes from preadipocytes) (Figure 4). Although adipocyte count is predominantly established early in life, recent research indicates that prolonged caloric surplus may stimulate the emergence of new adipocytes from preadipocytes, particularly in perivascular regions [62]. The equilibrium between hypertrophic expansion and adipogenesis significantly impacts metabolic well‐being. Interestingly, during overfeeding, subcutaneous AT of young, lean individuals expands through adipogenesis, but this capacity is diminished in individuals of advanced age and/or with obesity [60, 62].

Early evidence from the mid‐20th century correlates increased adipocyte size with systemic IR [63]. Conversely, small adipocytes are linked to mitigating obesity‐associated metabolic dysregulation (reduced insulin sensitivity and metabolic flexibility with adipose tissue dysfunction) and a reduced predisposition to diabetes. Hypertrophic adipocytes, as they enlarge, undergo mechanical stress and hypoxia, contributing to inflammation within AT. Larger adipocytes exhibit distinct biochemical properties, such as elevated lipolysis, increased secretion of inflammatory cytokines, and reduced production of anti‐inflammatory adipokines [62]. AT is classified into subcutaneous and visceral depots, each demonstrating distinct metabolic functions and responses to overnutrition. Numerous studies involving both humans and rodents have established a correlation between a higher proportion of subcutaneous AT compared with visceral AT and the preservation of metabolic health in individuals with similar body weight [64]. Thus, visceral AT is generally described as being primarily pro‐inflammatory and is more commonly linked with an increased risk of type 2 diabetes (T2D), hypertension, and dyslipidemia compared to subcutaneous AT [65]. Therefore, in obesity, the understanding of the pathophysiology of subcutaneous and visceral AT is relevant for managing and treating the condition effectively.

Considering these concepts, the AT expandability hypothesis (ATEH) posits that each individual inherently possesses a limit determining their capacity to store lipids in AT [66]. Once this limit is reached, AT loses its efficacy in lipid storage, resulting in the redirection of lipids toward alternative organs, such as the liver, muscle, pancreas, and kidneys. This phenomenon is called ectopic fat accumulation, and it is related to an increased risk of developing obesity complications. The precise mechanisms governing the limit on AT mass remain incompletely understood. As AT mass substantially increases in cases of obesity, cellular‐level responses include both adipocyte hyperplasia and hypertrophy.

### 4.1 CD36 in AT Expansion

As mentioned, adipocyte differentiation is crucial for healthy AT expansion during fuel oversupply. CD36 regulates adipocyte differentiation and adipogenesis [67], which implies that CD36 plays an important role in ATEH, which in turn is related to ectopic fat accumulation. Inhibition of the CD36 gene in preadipocytes disrupts their maturation, hampering the growth and development of AT [67]. Moreover, CD36 serves as an indicator of precursor cells in AT with a notable propensity for adipogenesis, likely by enhancing the absorption of lipids [68]. During AT expansion, the absence of CD36 has an adverse impact on the recruitment of preadipocytes to form adipocytes, and in fully mature adipocytes, the lack of CD36 is linked to increased basal breakdown of stored fats and enhanced sensitivity to insulin [69].

By measuring the expression of CD36 in visceral AT from individuals with and without obesity, Xiaoxiao Luo et al. showed that obesity triggers an increase in CD36 expression in preadipocytes, leading to lysosomal dysfunction and inflammation [36]. This process involves CD36 facilitating excessive calcium transfer from the endoplasmic reticulum to lysosomes through inositol (1,4,5)‐trisphosphate receptor 1 (IP3R1) activation, thereby impairing lysosomal function and promoting inflammation.

When comparing subcutaneous vs. visceral AT, Bonen et al [70]. found that protein CD36 level is significantly increased in subcutaneous AT of overweight, obese, and individuals with T2D. This contrasts with visceral AT, where CD36 levels were elevated in lean and overweight subjects but showed no difference in obese and T2D groups. The findings suggest a stronger correlation between CD36 expression in subcutaneous tissue and metabolic disturbances like elevated BMI and altered glucose and insulin levels, indicating a tissue‐specific response to metabolic health.

Taken together, these data indicate that CD36 displays dose‐ and state‐dependent actions in preadipocytes. Under physiological conditions, basal CD36 expression facilitates adipogenic commitment and recruitment, such that loss of CD36 impairs differentiation [67, 68, 69]. By contrast, in people with obesity/inflammatory context, obesity‐driven CD36 upregulation in early preadipocytes perturbs lysosomal Ca^2+^ homeostasis via IP3R1, induces lysosomal dysfunction and inflammation, and ultimately undermines healthy adipogenesis [36]. Accordingly, both deficiency and pathological overexpression can be harmful, suggesting a U‐shaped relationship between CD36 levels and preadipocyte function. This framework may also help reconcile depot‐specific patterns of CD36 reported in human adipose tissue [70].

Interestingly, the alterations in the lymphatic lipid transport system are linked to obesity and T2D. Loss of CD36 in lymphatic endothelial cells (LECs) in murine models results in lymphatic leakage, increased accumulation of visceral AT, and impaired glucose tolerance [71]. Apigenin, a compound found abundantly in various fruits and vegetables, demonstrates notable effects on body weight and the distribution of AT in mice subjected to HFD [72]. Specifically, it exerts a significant reduction in visceral AT while not significantly affecting subcutaneous AT and epididymal AT. The mechanism underlying this effect involves the downregulation of the expression of CD36. The decreased expression of CD36 within adipocytes subsequently leads to a reduction in the expression of PPAR‐γ, a crucial nuclear factor involved in adipogenesis [72]. Finally, using an in vitro approach with human adipocytes, recent work demonstrated that the axis FABP4/CD36 controls fat mass expandability (adipocyte size and number) [73], a crucial phenomenon for AT growth in obesity.

Overall, the evidence listed in this section suggests a direct relationship between CD36 regulation in obesity, adipocyte differentiation, and ATEH in terms of the subcutaneous vs. visceral fat storage model. CD36 supports adipogenic commitment and healthy recruitment under physiological conditions, whereas obesity‐driven CD36 upregulation in early preadipocytes perturbs lysosomal Ca^2+^ homeostasis and fosters inflammation, undermining adipogenesis. The evidence points to a dose‐ and state‐dependent (U‐shaped) relationship between CD36 and preadipocyte function. Key open questions include depot specificity in humans and how lymphatic and stromal compartments modulate CD36‐driven expandability.

### 4.2 Tissue‐Specific CD36 Regulation and Ectopic Lipid Deposition

Throughout this review, we adopt the updated steatotic liver disease (SLD) nomenclature: metabolic dysfunction‐associated steatotic liver disease (MASLD; formerly NAFLD) and metabolic dysfunction‐associated steatohepatitis (MASH; formerly NASH), per the 2023 multi‐society consensus and subsequent guidelines [74].

Obesity complications include a higher risk of ectopic fat accumulation in muscle, liver, heart tissue, pancreas, and kidney [75]. In turn, storing fats in these organs implies an increased risk of developing metabolic alterations, including IR, T2D, MASLD, and cardiovascular events [76]. Different studies have related CD36 activity to ectopic fat accumulation. When FA supply exceeds the need of the tissues, as observed in individuals with obesity who exhibit elevated circulating levels of non‐esterified fatty acids (NEFA), the normal regulation of CD36 is compromised. This impairment contributes to the ectopic accumulation of fat and a decline in mitochondrial function, culminating in a decrease in lean body mass and augmentation of fat mass, notably within the visceral AT depot [77].

CD36 regulation in the liver and skeletal muscle frequently relies on the metabolic state. Elevated expression of CD36 commonly occurs in reaction to high levels of insulin in the blood, potentially resulting in enhanced absorption of lipids in conditions of IR [78]. This increased lipid uptake subsequently contributes to the buildup of fat in unusual locations within these tissues, along with related metabolic dysfunctions [37, 79]. Notably, this phenomenon may not apply when FAs are acquired via LPL in skeletal muscle, as insulin appears to enhance LPL activity primarily in AT rather than in skeletal muscle [39].

Individuals with obesity have an increased risk of developing MASLD, in which CD36 in hepatocytes directly affects FAs uptake to promote MASLD development [40, 41]. Clinical evidence has pointed to the significance of CD36 by showing increased expression in the liver of patients with MASLD [42]. In the kidney, CD36 is highly expressed in tubular epithelial cells, mesangial cells, and podocytes. In obesity‐induced chronic kidney disease, CD36 is upregulated in the renal tissue, contributing to kidney fat accumulation [80]. In a cardiac context, increased adiposity in obesity is the strongest risk factor for developing diabetes and, in consequence, diabetic cardiomyopathy [81]. Increased levels of CD36 have been found in cardiac tissue from patients with diabetic cardiomyopathy, consequently exacerbating cardiac function [82]. A substantial influx of FAs is triggered by this elevated presence of CD36 in the sarcolemma. FAs present in the cytoplasm have the potential to stimulate PPAR, thus prompting the elevation of requisite enzymes crucial for mitochondrial β‐oxidation, consequently inducing a notable augmentation in FA oxidation rates. Nevertheless, the pace of FA absorption and retention surpasses that of oxidation, culminating in the buildup of lipids within the cardiac tissue [82].

The relationship between CD36 and fat accumulation in the pancreas, especially in obesity, is deeply intertwined with the role of CD36 in lipid metabolism and insulin signaling within pancreatic β‐cells. The increase in CD36 expression in the pancreas during obesity is linked to enhanced lipid availability and systemic IR [33]. Obesity increases circulating FFAs from heightened lipid mobilization and dietary intake, prompting upregulation of CD36 to facilitate FA uptake into pancreatic cells [33]. This is seen as an adaptive response of β‐cells to meet elevated metabolic demands for insulin production in states of IR, common in obesity. Systemic low‐grade inflammation in obesity can upregulate CD36 across multiple tissues via inflammatory mediators; here we highlight pancreatic β‐cell–specific effects, while a general overview is provided above in the section “CD36 in inflammation and IR in obesity” [83]. Increased CD36 expression impacts lipid metabolism and insulin signaling within β‐cells, enhancing FA absorption and promoting lipid accumulation, which impairs insulin secretion and β‐cell functionality [33, 83, 84].

**FIGURE 5:**
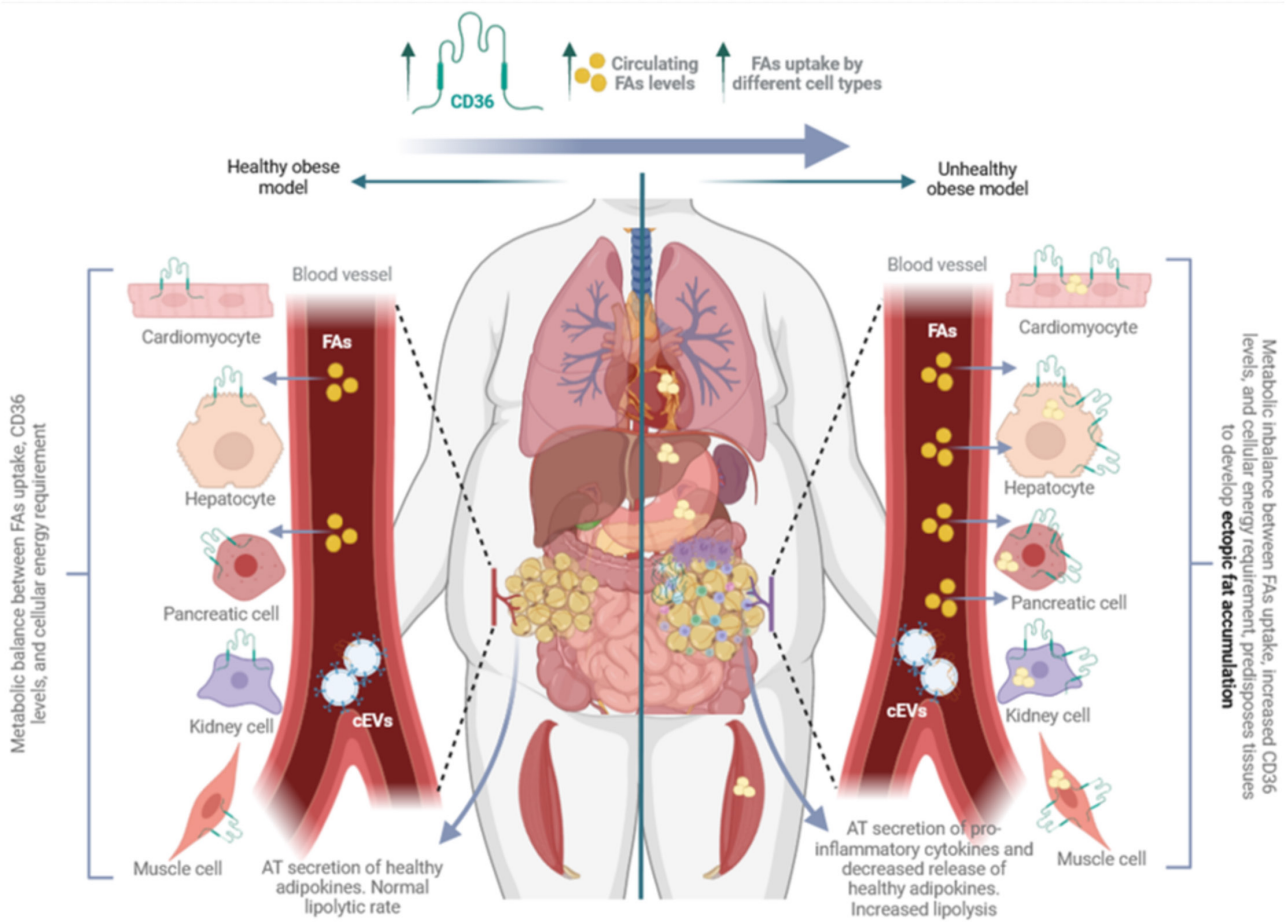
CD36 in healthy vs. unhealthy fat storage model. Individuals with obesity with normal levels of CD36 across different tissues show healthy metabolism indicators (circulating glucose and FAs) and fat is stored in the AT, known as the healthy obesity model. When AT storage capacity is exceeded and hypertrophic growth arises, secretion of pro‐inflammatory cytokines and decreased release of healthy adipokines is observed. Lipolysis rates are increased, which contributes to high levels of circulating FAs, which impacts the IR phenotype. The combination of high circulating levels of FAs with the increased amount of CD36 in different tissues, including cEVs, facilitates the lipid uptake contributing to developing ectopic fat accumulation in the unhealthy obesity model.

To summarize, the regulation of CD36 in different tissues is a crucial factor in developing ectopic fat deposition (Figure 5). In obesity, elevated NEFA and hyperinsulinemia increase CD36 abundance/trafficking in the liver, muscle, heart, kidney, and pancreas, amplifying FA uptake and contributing to steatosis and organ dysfunction. While this may begin as an adaptive response to metabolic demand, uptake outpaces oxidation, favoring lipid storage and lipotoxic stress. Unresolved issues include the relative roles of LPL‐derived vs. albumin‐bound FA pathways in muscle and the tissue‐specific thresholds at which CD36 shifts from adaptive to maladaptive.

## 5 CD36 in Extracellular Vesicles and General Metabolism

Extracellular vesicles (EVs) are a heterogeneous population of cellular‐derived particles that differ in size, content, and function [85]. According to new guidelines, EVs are classified based on their size into two main categories: small EVs (< 200 nm) and large EVs (> 200 nm) [86]. EVs are secreted by all cell types, such as adipocytes, and can be found in various body fluids including blood (circulating EVs: cEVs), urine, bile, and cerebrospinal fluid. EVs are known as critical players in cell‐to‐cell communication via direct surface interactions with target cells and/or delivery of intravesicular cargo since they carry various biomolecules [85]. The participation of EVs in human metabolism has been well documented during the last 15 years. EVs play a critical role in inter‐tissue communication and, in obesity, EVs establish crosstalk mechanisms between key tissues such as AT, muscle, liver, and brain [87]. It is known that adipose‐derived EVs (AT‐EVs) are major players in the development and progression of metabolic diseases such as obesity, diabetes, and IR [88, 89]. AT‐EVs can regulate liver expression of fibroblast growth factor 21 (FGF‐21) via delivery of microRNA [90] and increase insulin secretion through the transport of insulinotropic protein cargo [91]. CD36 can be expressed on the surface of EVs and we investigated the role of CD36 on cEVs and its relation to general metabolism. Thus, we showed that cEVs can uptake FFAs from serum and deliver them into heart tissue in a CD36‐dependent way [92]. Interestingly, CD36 is overexpressed on AT‐cEVs' surface isolated from HFD mice [88]. In adipocytes treated with palmitic acid, CD36 is sorted into EVs, and CD36‐expressing EVs are then endocytosed by hepatocytes to induce lipid accumulation and inflammation [93]. Regulation of tissue FA uptake is orchestrated by CD36 on endothelial cells (ECs). The subsequent division of caveolae produces EVs carrying FAs, CD36, and ceramide. These EVs are released basolaterally. In murine models, the inhibition of EV generation diminishes FA uptake in muscle tissues, elevates circulating FAs that persist within blood vessels, and reduces glucose levels, recapitulating key phenotypes observed in CD36−/− mice [94].

Interestingly, circulating levels of a soluble CD36 (sCD36) are elevated in patients with MASLD [95]. Collectively, findings by us and others investigating different types of circulating CD36 have shown considerable association with obesity and related complications. We developed an in‐house ELISA for measuring circulating sCD36, which has been used in numerous studies. Firstly, elevated levels of sCD36 are related to body fat distribution, BMI, and the amount of visceral and subcutaneous fat, FA uptake, and the level of liver damage observed in MASLD [96, 97, 98, 99, 100]. Secondly, sCD36 is strongly associated with obesity and the risk of T2D [96, 101, 102], and has been suggested as a predictor of T2D [103, 104]. Thirdly, levels of sCD36 are associated with arteriosclerosis risk in patients with T2D [105, 106, 107], and relate to the risk of stroke in severe arteriosclerosis [108]. Notably, sCD36 exhibits a U‐shaped association with liver fat, carotid intima–media thickness (IMT), and insulin resistance, consistent with a bimodal sCD36 distribution and increased risk at both very low and high levels [98]. Currently, it is not known to which degree this sCD36 form is expressed on cEVs, or if sCD36 corresponds to an alternative freely circulating isoform. Finally, we found increased levels of muscle‐derived CD36‐EVs post‐exercise in T2D [109], while CD36‐expressing EVs from monocytes were suggested as potential biomarkers for atherosclerosis [110]. Moreover, we showed that circulating monocyte‐derived CD36‐expressing EVs were reduced after bariatric surgery [111], and correlated with BMI, waist circumference, fat percentage, and indicators of hepatic fat accumulation [112]. Circulating levels of CD36 clearly associate with several obesity‐related complications and diseases, but also play an active role in these by regulating central inflammatory and metabolic pathways.

Circulating sCD36, and CD36‐positive EVs, associate with obesity, MASLD, T2D, and atherosclerosis, suggesting both biomarker value and potential mechanistic roles in lipid and inflammatory signaling. A central controversy is the biochemical provenance of ‘sCD36’ (EV‐bound versus soluble isoforms) and the need for standardized EV phenotyping to resolve causality versus correlation.

## 6 CD36 Genetics in Obesity

Current advancements in unbiased human genetic research, such as genomewide association studies (GWAS) and genomic methodologies like RNA‐sequencing, enable the exploration of the entire genome to uncover potential therapeutic genes [113, 114]. Previous research has associated CD36 polymorphisms with abnormal FFA levels and low‐density lipoproteins (LDL) [115, 116]. When studying isolated populations, different works have identified different CD36 genetic variants with the predisposition to develop metabolic diseases such as obesity. Several common CD36 SNPs, characterized by minor allele frequencies exceeding 5%, have been linked to various metabolic traits and complications related to obesity [117]. A study conducted by Noel S et al. investigated the role of CD36 in the metabolic syndrome (MetS) among Puerto Ricans, a population with a high prevalence of obesity. In this study, previously published CD36 SNPs were tested for association using multivariate linear regression models. Subjects homozygous for the rs1049673 G‐allele exhibited an increased risk of MetS [118]. The CD36 rs3211938 TG genotype demonstrates a correlation with elevated concentrations of glucose, oxidized low‐density lipoprotein (ox‐LDL), high‐density lipoprotein cholesterol (HDL‐C), IRIR, and an elevated BMI in individuals of Mexican mestizo descent diagnosed with T2D hailing from the western region of Mexico [119]. The rs3173798 genetic variation demonstrated a correlation with elevated rates of obesity and diabetes in the Caucasian population, increased levels of high‐sensitive C‐reactive protein (hs‐CRP), diminished serum concentrations of lipoprotein (a), and earlier onset of myocardial infarction among individuals [120]. Bokor et al., proposed that four CD36 genetic variants (rs3211867, rs3211883, rs3211908, and rs1527483) may be linked to fluctuations in body mass among adolescents of European descent [121]. Nevertheless, Choquet et al., by analyzing a cohort of 9973 subjects with similar ancestry, were not able to confirm the association of those variants with early‐onset obesity [122]. In a study of 1790 German subjects the association between four genetic variants (rs9784998, rs3211883, rs3211908, and rs3211956) within the CD36 gene and BMI was found to be statistically significant, as well as the association of rs3211883 and rs3211908 with waist circumference. Conversely, the variants rs3211816 and rs3211960 within CD36 showed no significant correlation with adiposity measures. It is plausible that genetic diversity within the CD36 gene locus could influence metabolic disorders primarily through its impact on body fat accumulation [123]. The allele ‐22674C within the CD36 promoter region is correlated with reduced levels of low‐density lipoprotein‐cholesterol (LDL‐C) in UK female twins within the normal range and with enhanced lipid profile progression during the process of weight reduction and maintenance among individuals with obesity [115].

Overall, it is evident that CD36 genetics contributes to the diversity observed in lipid profiles among individuals and their susceptibility to complications related to obesity. The available evidence concerning CD36 genetics and outcomes pertinent to health is restricted, necessitating further investigations in functional genomics to validate its role and elucidate its mechanism of action. Exploring the association between CD36 gene variants and diseases will facilitate a deeper comprehension of CD36 gene ontology, protein functionality, and the pathophysiological underpinnings of associated conditions. Comparative studies involving individuals deficient in CD36 [124, 125, 126, 127, 128] versus those exhibiting diminished CD36 levels [129] due to common CD36 single SNPs suggest that reduced CD36 expression confers metabolic protection. Conversely, complete absence of CD36, akin to CD36 overexpression, is likely to predispose individuals to metabolic complications. This implies the existence of a potential “metabolically protective” range concerning CD36 expression.

## 7 Perspective

The comprehensive review of CD36's role across various metabolic and physiological contexts underlines its pivotal function in the etiology of obesity and its complications. This integral membrane protein, beyond its primary role in FA transport, emerges as a crucial player in metabolic regulation. The multifunctional nature of CD36, interacting within complex metabolic pathways, presents interesting opportunities for therapeutic interventions aimed at mitigating obesity‐related complications.

Clinical trials have demonstrated the effectiveness of weight loss, achieved through either dietary modifications or surgical intervention [130], in reducing obesity and its associated comorbidities [131, 132]. Despite the proven benefits, sustaining long‐term adherence to dietary regimens remains a challenge [133]. The pursuit of durable pharmacological interventions presents a potential solution to the challenges posed by adherence issues and the morbidity associated with surgical procedures. Unfortunately, the history of weight loss drugs is complex with instances where products have successfully induced weight loss but at the expense of causing clinical harm [134]. New approaches are needed to treat and manage obesity, and CD36 as an important player in AT biology, represents an interesting target to develop therapeutic strategies.

Converging genetic and pharmacological evidence supports CD36 as a target to mitigate ectopic lipid deposition and downstream metabolic dysfunction. Causality is established by hepatocyte‐specific CD36 deletion, which protects mice from high‐fat‐diet‐induced steatosis and improves insulin sensitivity, validating hepatocellular CD36 as a driver of MASLD [40]. Translationally, peripherally restricted CB1 antagonism has re‐emerged as a clinically plausible upstream route to “dial down” hepatic CD36: in diet‐induced obesity, peripheral CB1 blockade reverses steatosis while reducing hepatic CD36 expression and restoring fatty‐acid oxidation, without central CB1 liabilities [135]. Osteoprotegerin (OPG) promotes hepatosteatosis via an ERK‐PPARγ‐CD36 cascade; PPARγ inhibition or ERK activation prevents OPG‐driven CD36 induction, and OPG fails to drive lipid accumulation when CD36 is absent—nominating this axis for indirect CD36 suppression [136]. Extrahepatic levers are complementary. In the intestine, AMPK regulates CD36 abundance and membrane translocation in enterocytes; intestinal AMPK deletion lowers CD36, limits long‐chain fatty‐acid uptake, and protects against diet‐induced obesity—implicating gut‐selective AMPK–CD36 manipulation as an adjunct strategy, with attention to tissue specificity [30]. In parallel, noncoding RNA therapeutics offer potential control of CD36: miR‐133a reduces lipid loading by repressing TR4‐CD36 signaling in macrophages [137]; miR‐758‐5p directly targets the CD36 3′UTR to curb cholesterol uptake [138]; and cardiomyocyte miR‐100 overexpression lowers CD36 and fatty‐acid uptake, illustrating tissue‐directed CD36 down‐tuning [139]. The TINCR/miR‐107/CD36 axis further exemplifies post‐transcriptional CD36 control relevant to proliferative and metabolic programs [140], while immune–metabolic crosstalk through miR‐375/CD36 underscores how microRNAs can remodel macrophage phenotype and lipid handling with potential relevance to hepatic inflammation [141]. Together, these data support a therapeutic approach to reduce ectopic fat and inflammation. However, the therapeutic targeting of CD36 is challenging. The protein's ubiquitous expression and essential roles in various tissues necessitate precise, context‐dependent modulation to avoid unintended systemic effects.

In addition to pharmacological approaches, the manipulation of dietary patterns presents another intriguing avenue for modulating CD36's activity. Emerging evidence suggests that dietary components can influence CD36 expression and function, offering a dietary intervention strategy to manage obesity and its complications. Regarding CD36 genetics and types of fat consumption, Zheng et al., demonstrated that patients with T2D with different CD36 genotypes respond differentially to the intervention of omega‐3 supplements in blood lipid profiles [142]. Variations in the CD36 gene have been linked to regular intake of dietary fats, potentially influencing subsequent correlations with biomarkers indicative of chronic diseases. These associations exhibit disparities based on individuals' BMI status and the specific types of fats consumed in their diets [143]. In the same line of evidence, work published by Burgess et al., showed that CD36 genetic variants may have ethnic‐specific effects on fat taste perception [52]. Genetic signals identified by GWAS do not offer immediate insight into the mechanisms underlying disease causation [113]. Moreover, a critical limitation of GWAS is the demographic bias [144], and new research is needed to elucidate those limitations. Taken together, the findings mentioned in this section, establish a direct relation between dietary fat and CD36 genetics with metabolic outcomes. The general metabolic homeostasis through CD36 mediation faces controversy due to the presence of two main variability factors across populations: dietary habits and genetics. The impact of polyunsaturated fatty acids (PUFA) on CD36‐mediated cholesterol homeostasis remains a subject of study, contingent upon the quantities of n‐3 PUFA, n‐6 PUFA, and the n‐3/n‐6 PUFA ratio [145]. Nevertheless, while new research is needed to better understand these points, the idea of tailoring diets to modulate CD36 activity could complement pharmacological strategies, providing a holistic approach to managing obesity in personalized medicine.

In conclusion, the study of CD36 stands as a beacon guiding us toward a more comprehensive knowledge of obesity's molecular foundation. However, our journey to fully elucidate CD36's role in obesity is far from complete. As we advance our understanding, it is crucial that our strategies evolve from mere treatment to include prevention, integrating dietary, pharmacological, and behavioral interventions that are tailored to individual metabolic profiles and genetic predispositions.

## Conflicts of Interest

The authors declare no conflicts of interest.

## Data Availability

Data sharing not applicable to this article as no datasets were generated or analysed during the current study.
